# Inhibition of Microbicidal Activity of Canine Macrophages DH82 Cell Line by Capsular Polysaccharides from *Cryptococcus neoformans*

**DOI:** 10.3390/jof10050339

**Published:** 2024-05-08

**Authors:** Isabel F. LaRocque-de-Freitas, Elias Barbosa da Silva-Junior, Leticia Paixão Gemieski, Beatriz da Silva Dias Lima, Israel Diniz-Lima, Aislan de Carvalho Vivarini, Ulisses G. Lopes, Leonardo Freire-de-Lima, Alexandre Morrot, José Osvaldo Previato, Lucia Mendonça-Previato, Lucia Helena Pinto-da-Silva, Celio G. Freire-de-Lima, Debora Decote-Ricardo

**Affiliations:** 1 Instituto de Veterinária, Universidade Federal Rural do Rio de Janeiro, Seropédica 23890-900, Brazil; belarocque@gmail.com (I.F.L.-d.-F.); leticiagemiesk@gmail.com (L.P.G.); beatrizdias@ufrrj.br (B.d.S.D.L.); lpinto@ufrrj.br (L.H.P.-d.-S.); 2 Instituto de Biofísica Carlos Chagas Filho, Universidade Federal do Rio de Janeiro, Rio de Janeiro 21941-902, Brazil; eliasbarbosa@biof.ufrj.br (E.B.d.S.-J.); israel@biof.ufrj.br (I.D.-L.); lopesu@biof.ufrj.br (U.G.L.); leolima@biof.ufrj.br (L.F.-d.-L.); previato@biof.ufrj.br (J.O.P.); luciamp@biof.ufrj.br (L.M.-P.); 3 Instituto de Biologia, Universidade Federal Fluminense, Niterói 22220-900, Brazil; aislanvivarini@gmail.com; 4 Instituto Oswaldo, FIOCRUZ, Rio de Janeiro 21045-900, Brazil; alexandre.morrot@ioc.fiocruz.br; 5 Faculdade de Medicina, Universidade Federal do Rio de Janeiro, Rio de Janeiro 21941-900, Brazil

**Keywords:** canine macrophages, capsular polysaccharides, inhibition, cryptococcosis

## Abstract

*Cryptococcus neoformans* is a lethal fungus that primarily affects the respiratory system and the central nervous system. One of the main virulence factors is the capsule, constituted by the polysaccharides glucuronoxylomannan (GXM) and glucuronoxylomanogalactan (GXMGal). Polysaccharides are immunomodulators. One of the target cell populations for modulation are macrophages, which are part of the first line of defense and important for innate and adaptive immunity. It has been reported that macrophages can be modulated to act as a “Trojan horse,” taking phagocytosed yeasts to strategic sites or having their machinery activation compromised. The scarcity of information on canine cryptococcosis led us to assess whether the purified capsular polysaccharides from *C. neoformans* would be able to modulate the microbicidal action of macrophages. In the present study, we observed that the capsular polysaccharides, GXM, GXMGal, or capsule total did not induce apoptosis in the DH82 macrophage cell line. However, it was possible to demonstrate that the phagocytic activity was decreased after treatment with polysaccharides. In addition, recovered yeasts from macrophages treated with polysaccharides after phagocytosis could be cultured, showing that their viability was not altered. The polysaccharides led to a reduction in ROS production and the mRNA expression of IL-12 and IL-6. We observed that GXMGal inhibits MHC class II expression and GXM reduces ERK phosphorylation. In contrast, GXMGal and GXM were able to increase the PPAR-γ expression. Furthermore, our data suggest that capsular polysaccharides can reduce the microbicidal activity of canine macrophages DH82.

## 1. Introduction

Cryptococcosis is a systemic disease that affects animals and people worldwide, caused by an encapsulated yeast species of the genus Cryptococcus with a predilection for the nervous and respiratory systems [1]. The infection in dogs is frequently caused by *Cryptococcus neoformans* and *Cryptococcus gattii*. Clinical signs depend on the sites of infection, but the involvement of critical organs, such as the central nervous system (CNS), eyes, gastrointestinal tract, myocardium, adrenal glands, and pancreas, in dogs with cryptococcosis is frequently observed [2]. Even though the reported incidence of cryptococcosis is lower in dogs than in cats, CNS involvement may be more common in dogs, with widespread dissemination to other organs [3]. Macrophages are crucial for cryptococcosis control. It has been demonstrated that macrophage and dendritic cell depletion significantly reduced mice survival after infection with *C. neoformans*. Additionally, the Trojan horse hypothesis suggests that a fungus enters into the blood–brain barrier through dissemination within the macrophage [4,5]. In this context, for *C. neoformans*, a Trojan horse mechanism for dissemination is supported by the results obtained after depletion of alveolar macrophages, preventing brain dissemination [6]. In addition, an increased brain fungal burden was observed in experiments with mice who were injected with ex vivo infected macrophages [7]. Although numerous proteins are important in interaction with macrophages [8], capsular polysaccharides have shown an important ability to deactivate the immune system [9]. The capsular polysaccharides of *C. neoformans* are the main virulence factor. The capsule is composed of glucuronoxylomannan (GXM), glucuronoxylomanogalactan (GXMGal), and small amounts of mannoproteins. GXM represents 90% of the capsule, GXMGal around 8%, and mannoproteins have been identified (2%) but have not been studied in detail yet [10]. Previous studies have demonstrated that capsular polysaccharides such as GXM and GXMGal were able to induce impairment of macrophage activity, demonstrated by the modulation of pro-inflammatory cytokines, apoptosis induction, and modulated production of neutrophil extracellular traps (NETs) by human neutrophils [11,12]. GXM can be captured by different receptors, particularly Toll-like receptors (TLRs), CD14, CD18, FcγRIIB, CD18, dectin, and manose receptors [13,14]. The adapter protein associated with TLR, the MyD88 molecule, is known to play an important role in the immune response, since animals who are deficient in MyD88 showed an increased susceptibility to cryptococcosis [14,15]. The capsular polysaccharides of *C. neoformans* are important pathogen-associated molecular patterns that are essential for stimulating cellular immunity. However, there is no cellular receptor that specifically recognizes these polysaccharides. Microorganism–host recognition can occur through numerous receptors that are present on the cell surface [16]. Inside the macrophage, GXM produces multiple effects, for example, reduction in the antigen-presenting cell (APC) function [17], increased FasL, and deregulation of pro-inflammatory and anti-inflammatory secretion of cytokines. Indeed, low doses of GXMGal can induce FasL expression and inhibit proliferation in macrophage cell lines, indicating that GXMGal is more potent for producing immunomodulatory effects [11]. Macrophages from different species have some peculiarities. Canine and murine genomes differ significantly. This dissimilarity can lead to variances in the expression of genes involved in macrophage function, such as pattern recognition receptors (PRRs), cytokines, and chemokines, and may lead to variances in their response to stimuli. The regulatory mechanisms governing the activation and modulation of macrophage activity may differ between species. Macrophages exhibit functional diversity depending on their polarization state, with M1 macrophages being pro-inflammatory and M2 macrophages being anti-inflammatory. However, traditional markers for distinguishing M1 and M2 macrophages in humans and mice, such as CD16, CD32, iNOS, CD163, and arginase-1, were not effective in discriminating canine macrophage phenotypes, and only CD206 was able to discriminate M2 macrophages from M0 and M1 phenotypes, highlighting this molecule as a promising marker for canine M2 macrophages. Transcriptomic analysis demonstrated a relatively low overlap between mice and human gene expression profiles associated with M1 and M2 polarization in canine macrophages, suggesting species-specific differences in macrophage polarization. Pathway analysis revealed enrichment in the metabolic pathways for M1 macrophages and peroxisome proliferator-activated receptor (PPAR) signaling for M2 macrophages [18]. Most of the data in the literature refer to analyses carried out on mouse macrophages, where polysaccharides seem to exert an immunomodulatory effect in several effective functions [8,11,19,20]. However, there is a gap in understanding whether these effects that are observed in murine macrophages are the same in canine macrophages. There are no reports on the possible effects of these polysaccharides on canine macrophages. Knowledge of these mechanisms may bring light on the understanding of the initial moments of the interaction with *C. neoformans*. Therefore, in this study, the objective was to evaluate the ability of the purified capsular polysaccharides from *C. neoformans* to modulate the activity of the canine macrophage cell line DH82.

## 2. Materials and Methods

### 2.1. DH82 Cell Line Culture

Canine macrophages DH82 (ATCC) were cultured in 75 cm^2^ tissue culture flasks (T75) (Nunc, Roskilde, Denmark) with Dulbecco’s Modified Eagle Medium (DMEM; Sigma-Aldrich) supplemented with 10% fetal calf serum (FCS; Gibco), 1% MEM non-essential amino acids (Sigma-Aldrich, Saint Louis, MI, USA), 100 μg/mL streptomycin, 100 Units/mL penicillin, and 2 mM L-glutamine. Cells were maintained at 37 °C with 5% CO_2_. Subculturing was performed weekly upon reaching 95–100% confluence. Cells were detached using 0.25% trypsin (Difco, Franklin Lakes, NJ, USA) and 1 mM EDTA (Sigma-Aldrich), harvested, and washed twice in Hank’s Balanced Salt Solution (HBSS) by centrifugation at room temperature for 10 min at 250 g. After discarding the supernatant, the cell pellet was resuspended in DMEM containing 10% FCS and cultured in new T75 flasks. The culture medium was changed after 3 days, and cell viability was assessed using trypan blue exclusion before use in all assays.

### 2.2. Isolation and Purification of C. neoformans Capsular Polysaccharides (GXMGal and GXM)

The isolation and purification of polysaccharides were performed as previously described [19]. GXM was obtained from the culture supernatant of the encapsulated strain B-3501, while GXMGal was obtained from the culture supernatant of the Cap67 strain lacking GXM. Briefly, cells grown in the defined liquid medium at 37 °C with continuous shaking (100 rpm) for 5 days were removed by centrifugation (12,000× *g* for 1 h at 4 °C), and the capsular polysaccharides in the supernatant were precipitated after adding three volumes of cold ethanol. To separate GXMGal from mannoproteins, the capsular polysaccharides were fractionated using lectin-affinity chromatography on a XK-26 column (Pharmacia, Stockholm, Sweden) packed with 70 mL of Concanavalin A-Sepharose 4B (Pharmacia) at 4 °C. The unbound polysaccharide fractions, containing GXMGal, were localized through a phenol-sulfuric reaction, pooled, dialyzed against distilled water, and lyophilized. Pure GXMGal was obtained through anion-exchange chromatography on a MonoQ (HR16/10) column using a 50 mL super loop. GXM was purified by differential precipitation with cetyltrimethyl ammonium bromide (CTAB). Capsular polysaccharides isolated from the culture supernatant by precipitation with ethanol were dissolved in 0.2 M NaCl (10 mg/mL), and CTAB (3 mg/mg of polysaccharides) was added slowly. A solution of 0.05% CTAB was then added, and GXM was selectively precipitated. The precipitate was collected by centrifugation, washed with 2% acetic acid in ethanol, and then washed in 90% ethanol. The precipitate was dissolved in 1 M NaCl, and three volumes of ethanol were added to precipitate the GXM. After centrifugation and washing with 2% acetic acid in ethanol, the precipitate was dissolved in water and lyophilized. To eliminate potential lipopolysaccharide (LPS) contamination, 10 mg of GXMGal or GXM preparations was dissolved in LPS-free water and further purified through chromatography on a column of Polymyxin B-Agarose (Sigma, Saint Louis, USA), equilibrated with LPS-free water. Purified GXMGal or GXM was eluted with 12 mL of LPS-free water, recovered, and lyophilized. The polysaccharides were dissolved in PBS before being added to macrophage cultures.

### 2.3. Apoptosis Assay

DH82 macrophages (1 × 10^5^) were seeded in a 48-well plate and cultured in a cell culture incubator at 37 °C with 5% CO_2_ using DMEM supplemented with 10% FCS, 1% MEM non-essential amino acids, 100 μg/mL streptomycin, 100 Units/mL penicillin, and 2 mM L-glutamine in 500 µL/well. After 24 h, the wells were washed, and fresh culture medium was added. GXMGal, GXM, and total capsule were added at concentrations of 10, 50, and 100 µg/mL for 24 h. Quadruplicate samples were prepared for each condition. After incubation, the wells were washed twice with PBS, and the cells were detached using 200 µL of 0.25% trypsin (Difco) and 1mM EDTA (Sigma-Aldrich) for 5 min. Trypsin activity was stopped by adding 100 µL of FCS. Staining and analysis were performed according to the manufacturer’s instructions (BD Pharmingen—Latin America (556547). The cells were centrifuged at 1600 RPM for 6 min in a U-bottom plate, resuspended, and labeled with 5 µL of FITC Annexin V solution (diluted 1:20 in 1x Binding Buffer provided by the manufacturer) and 5 µL of propidium iodide (PI) for 15 min at room temperature. The Binding Buffer (400 µL) was added, and the samples were protected from light and analyzed within 1 h using a flow cytometer BD LSRFortessa X20. Data were analyzed using FlowJo X software v10.10.

### 2.4. Assessment of Binding and Phagocytosis

DH82 cells were plated on glass coverslips at a density of 1 × 10^4^ cells/well and incubated overnight with different concentrations (10, 50, and 100 µg/mL) of GXM, GXMGal, and total capsule. The concentrations of polysaccharides used were based on our previous work [11,12]. Subsequently, *Saccharomyces cerevisiae* yeast was added to the cells at a ratio of 10:1 for 40 min (binding assay) or 4 h (phagocytosis assay) and incubated at 37 °C with 5% CO_2_. After incubation, the cells were washed twice with PBS to remove non-binding and non-phagocytosed yeasts. The coverslips were then washed with HBSS, fixed with methanol, and stained with Diff-Quick (Thermo Fisher, Waltham, MA, USA). The yeasts were counted at 100x oil immersion under an Olympus microscope. The number of yeasts was estimated in 100 cells per coverslip, and the frequency was compared among six coverslips per time point.

### 2.5. Viability of Yeasts after Phagocytosis

Canine DH82 macrophages were plated in 24-well culture plates at a density of 1 × 10^4^ cells/well and incubated with the capsular polysaccharides and total capsule for 24 h at the concentrations described above. After this period, *S. cerevisiae* yeasts were added at a ratio of 10:1 (yeasts/macrophage) and incubated for 4 h. Subsequently, the cultures were washed twice with PBS and lysed with ice water (500 µL). The lysate was diluted 1:10 in 1× PBS, and a 30 µL volume of the diluted lysate was seeded onto Petri dishes containing Sabouraud agar. The plates were incubated at 37 °C for 48 h, and the colony-forming units were counted [20].

### 2.6. Reactive Oxygen Species (ROS) Detection

The intracellular levels of ROS were quantified by the oxidation of the non-fluorescent 2′,7′-dichlorofluorescein probe, delivered in the diacetate form (DCFH-DA), to the fluorescent product 2′,7′-dichlorofluorescein [21]. DH82 macrophages were plated in 96-well plates at a density of 5 × 10^4^ cells/well, incubated with polysaccharides (GXMGal, GXM, and capsule) at a concentration of 100 µg/mL, stimulated or not with LPS (400 ng/mL, Sigma-Aldrich) and recombinant IFN-γ (1.5 ng/mL, Serotec, Oxford, UK), and after 24 h, the cells were washed and loaded with 10 μM DCFH-DA (Invitrogen, Waltham, MA, USA) for 20 min at 37 °C. Untreated and unstimulated cells were used as a negative control. After incubation, the cells were washed, and the fluorescence was measured (excitation = 485 nm, emission = 535 nm) using an FLx800 Fluorescence Microplate Reader (BioTek, Winooski, VT, USA).

### 2.7. Real-Time RT-PCR Quantification

DH82 macrophages (1 × 10^6^ cells) were plated and incubated with GXMGal, GXM, and capsule (100 µg/mL) and stimulated or not with LPS (400 ng/mL) and IFN-γ (1.5 ng/mL) for 24 h. Total RNA was extracted using the RNeasy Plus Mini Kit (Qiagen 74134, Hilden, Germany), and a 1 μg aliquot was reverse-transcribed to first-strand cDNA using ImProm-II (Promega, Madison, USA) and oligo(dT) primer according to the manufacturer’s instructions. The DNA sequences of the primers used were as follows: IL-6-F: 5′-GCGTCTTCCCTCATGACC-3′, IL-12-R: 5′-GGGTGCCAGTCCAACTCTAC-3′, IL-6-F:5′-GGGAAAGCAGTAGCCATCAC-3′,IL-6-R:5′CAGGACCCCAGCTATGAACT-3′,TGF-β-F:5′-CGAAGCCCTCGACTCC-3′,TGF-β-R:5′TGGCTGYCCTTTGATGTCAC-3′, GAPDH-F: 5′-TGCACCACCAACTGCTTAGC-3′ and GAPDH-R: 5′-GGCATGGACTGTGGTCATGAG-3′. qRT-PCR data were normalized using Gapdh primers as an endogenous control. The amplicon specificity was verified by the presence of a single melting temperature peak in dissociation curves, run after the real-time RT-PCR. Real-time quantitative RT-PCR (qRT-PCR) was performed using the Applied Biosystems StepOne™ detection system (Applied Biosystems, Waltham, MA, USA) with GoTaq^®^ qPCR Master Mix (Promega Corp., Madison, WI, USA). Expression ratios were computed via the analysis of the relative gene expression ΔΔCt method using StepOne software version 2.0 (Applied Biosystems). Standardization of all real-time PCR reactions, including primer concentration, melting curve analysis, and standard curve construction, was conducted to ensure efficiency across all targets. Analysis of relative expression in relation to the endogenous control GAPDH was performed after confirming similar Ct (“Cycle Threshold”) values among all samples tested.

### 2.8. MHC Class II Expression

The expression of the major histocompatibility complex class II (MHC class II) was evaluated in DH82 macrophages cultured in 6-well culture plates (1 × 10^6^ cells/well) and incubated for 24 h with the capsular polysaccharides (100 µg/mL), LPS (400 ng/mL), and IFN-γ (1.5 ng/mL). After 24 h, the cells were detached, washed, adjusted to a concentration of 5 × 10^5^ cells/tube, and incubated with a blocking buffer (CD16/CD32 Fc Block-eBioscience) for 15 min on ice to prevent the non-specific antibody binding to Fc receptors. Cells were then stained with anti-dog MHC class II-FITC (Serotech). All washing steps were performed with PBS containing 3% FCS and 0.02% sodium azide. Data were acquired (10,000 events), evaluated on a FACSCalibur™ cytometer, and analyzed using CellQuest^®^ software version 5.1 (BD Biosciences, Heidelberg, Germany).

### 2.9. Assay of Protein Phosphorylation

The DH82 macrophage cell lines were cultured in 6-well culture plates (1 × 10^6^ cells/well) and incubated for 24 h with the polysaccharides GXMGal, GXM, and total capsule (100 µg/mL) and stimulated with canine IFN-γ (1.5 ng/mL) and LPS (400 ng/mL). The Western blotting protocol has been described previously [22]. The primary antibodies used were anti-ERK (4696S—Cell Signaling, Danvers, MA, USA), anti-pERK (4370S-Cell Signaling, Danvers, USA), anti-PPAR-γ (2435—Cell Signaling, Danvers, USA), and anti-β actin (Sigma-Aldrich, Saint Louis, USA), with anti-IgG (whole molecule)-peroxidase (Sigma-Aldrich) used as a secondary antibody. Reactions were developed using a chemiluminescence kit (ECL Western Blotting Substrate-Promega-w1015) according to the manufacturer’s instructions. The band densitometry of the Western blotting was analyzed using Scion Image software 4.0.3.2.

### 2.10. Statistical Analysis

Data analysis was performed using GraphPad Prism v5.0. Results are expressed as mean and standard deviation (SD). All experiments were performed in triplicate as indicated in the figure legends. Data were compared using one-way analysis of variance (ANOVA), followed by Dunnett’s post-test. Significance was considered for * *p* < 0.05, ** *p* < 0.01, and *** *p* < 0.001.

## 3. Results

### 3.1. Purified Polysaccharides and Total Capsule Do Not Induce Apoptosis in DH82 Canine Macrophage Cell Lines

Capsular polysaccharides from *C. neoformans* have previously been described as inducers of apoptosis in murine macrophages [11]. Therefore, we initially investigated whether the purified polysaccharides would exhibit any toxic effects on canine macrophages of the DH82 cell line.

**Figure 1:**
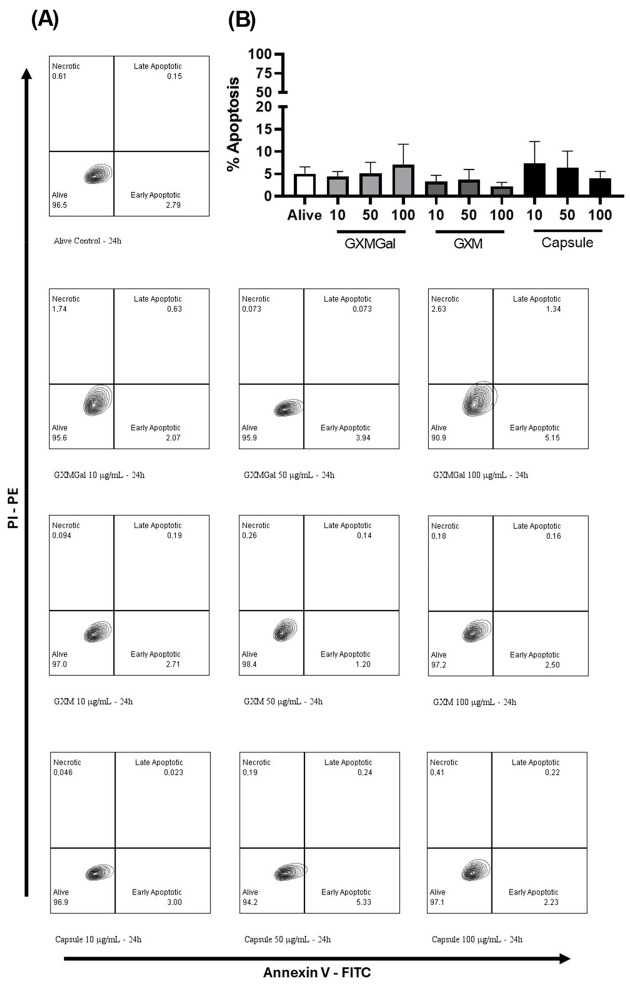
The effect of purified polysaccharides and the total capsule on the apoptosis induction of DH82 cells. Canine macrophage DH82 cultures were maintained at a concentration of 105 cells/well and incubated for 24 h. Subsequently, cells were treated with GXMGal, GXM, and the total capsule at the specified concentrations (μg/mL). Representative scatter plots of Canine macrophage DH82 under different conditions (**A**) and frequency of apoptosis induction by purified polysaccharides capsule in DH82 cells under the tested conditions (**B**). Alive cells represent macrophages without polysaccharide treatment. The data represent the mean ± SEM of three independent experiments, conducted in quadruplicate. Statistical analysis was performed using one-way ANOVA followed by Dunnett’s post-test.

For this purpose, DH82 macrophage cell cultures were established and treated with GXM-Gal, GXM, or *C. neoformans* total capsule at different concentrations, followed by a 24 h incubation period. Subsequently, the presence of apoptotic cells was quantified using an annexin V/PI staining kit. Our experiments revealed that neither the polysaccharides nor the total capsule induced apoptosis in DH82 macrophages after 24 h of incubation (Figure 1). Notably, there were no statistical differences observed between the groups, indicating that neither the purified polysaccharides nor the total capsule induced apoptosis in DH82 cells under the tested conditions (Figure 1).

### 3.2. Purified Polysaccharides from C. neoformans Inhibit Binding and Phagocytic Activity of Canine Macrophages DH82

The ability to bind to and phagocytose pathogens is crucial for the elimination of infectious agents [23]. Given that binding and phagocytosis are primary functions of macrophages, we initiated an investigation into the effects of DH82 cell contact with the polysaccharides GXM-Gal, GXM, or the total capsule on these mechanisms.

**Figure 2:**
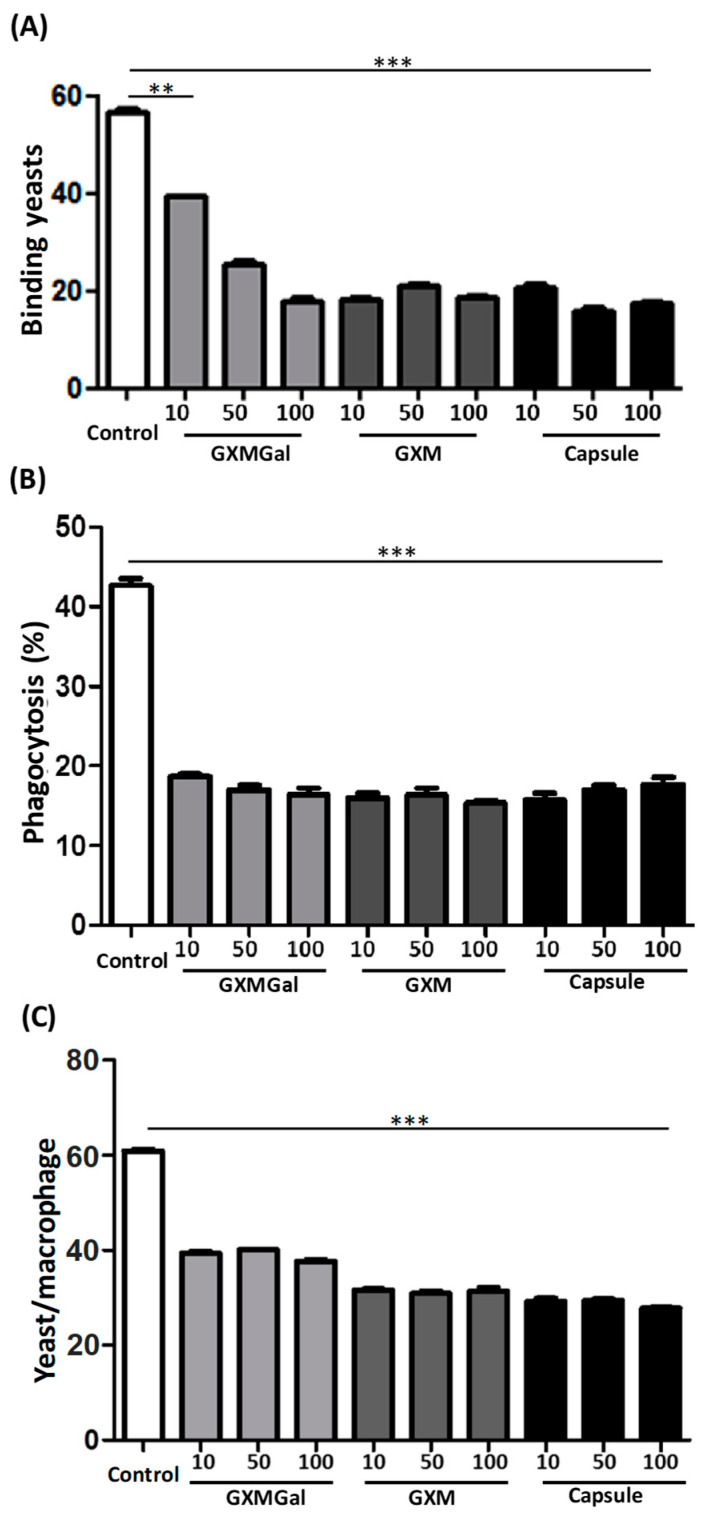
Binding and phagocytosis by DH82 in the presence of capsular polysaccharides of *C. neoformans*. DH82 cells were plated on a glass coverslip (1 × 10^4^ cells/well) and incubated with GXMGal, GXM, and capsule at the indicated concentrations (μg/mL). Control represents unstimulated samples not treated with polysaccharides. After 24 h, *S. cerevisiae* (10 × 10^4^ cells/well) was added to the culture. After taking 40 min for the evaluation of binding (**A**), we waited 4h to evaluate the percentage of cells that phagocytized (**B**) and number of phagocytosed yeasts (**C**). The cells were then stained, and the count was performed at 100x magnification. The data represent mean ± SEM of three independent experiments, conducted in triplicates. Statistical analysis was performed using one-way (ANOVA), followed by Dunnett’s post-test. We considered significant differences indicated for ** *p* < 0.01 and *** *p* < 0.001.

To assess the impact on binding and phagocytosis, DH82 cells were cultured in the presence of purified polysaccharides or the total capsule and subsequently incubated with S. cerevisiae yeasts for 40 min. Following incubation, we observed a reduction in the binding capacity of cells treated with polysaccharides, as evidenced by the decreased number of yeasts attached to the surface of DH82 cells (Figure 2A).

Similarly, a decrease in phagocytic activity was observed in DH82 cells treated with polysaccharides. This reduction was evident in both the number of cells that were capable of phagocytosing yeast (Figure 2B) and the quantity of yeast that was phagocytosed by macrophages (Figure 2C). Our findings suggest that exposure to C. neoformans polysaccharides may impair the ability of DH82 cells to bind to particles and subsequently undergo phagocytosis.

### 3.3. Yeasts Recovered from DH82 Macrophages Treated with Polysaccharides Show Increased Growth

Given the observed inhibition of phagocytosis in the presence of polysaccharides, we sought to determine whether the phagocytized yeasts remained viable and if potential macrophage modulation supported their survival.

DH82 cells were cultured and incubated with GXMGal, GXM, or the total capsule. Subsequently, S. cerevisiae was added to the culture and incubated for 4 h. Phagocytosed yeasts were then recovered after macrophage lysis and plated on Sabouraud agar, following a previously described protocol [20].

**Figure 3:**
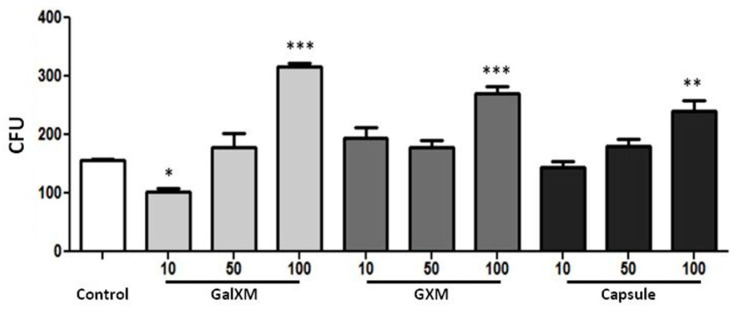
The fungicidal activity of canine macrophages DH82 treated with purified polysaccharides from *C. neoformans*. DH82 cell cultures were plated (1 × 10^4^ cells/well) and treated with GXMGal, GXM, or capsule at the indicated concentrations (μg/mL). Control represents unstimulated samples that were not treated with polysaccharides. Yeasts of *S. cerevisiae* (10 × 10^4^ cells/well) were added to the culture and incubated for 4 h. After incubation, the macrophages were lysed, and the recovered yeasts were seeded on Sabouraud agar and incubated for 3 days. After incubation, colony-forming units were counted. The data represent the mean ± SEM of three independent experiments, conducted in triplicates. Statistical analysis was performed by variance (ANOVA), followed by Dunnett’s post-test. We considered significant differences indicated for * *p* < 0.05, ** *p* < 0.01, and *** *p* < 0.001.

We observed a higher number of colony-forming units (CFUs) of yeasts recovered from DH82 cells that were treated with polysaccharides or the total capsule. Specifically, yeasts recovered from cells treated with *C. neoformans* polysaccharides at a concentration of 100 µg/mL exhibited significant growth compared to yeasts recovered from untreated cells (Figure 3). This suggests a potential alteration in the microbicidal mechanisms of canine macrophages, induced by the exposure to *C. neoformans* polysaccharides.

### 3.4. Polysaccharides from C. neoformans Inhibit the Production of Reactive Oxygen Species in Canine Macrophages DH82 Cell Line

Reactive oxygen species (ROS) production is a critical mechanism for macrophage microbicidal function. Hence, we investigated the impact of treatment with purified polysaccharides and the total capsule on the ROS production in DH82 macrophages.

**Figure 4:**
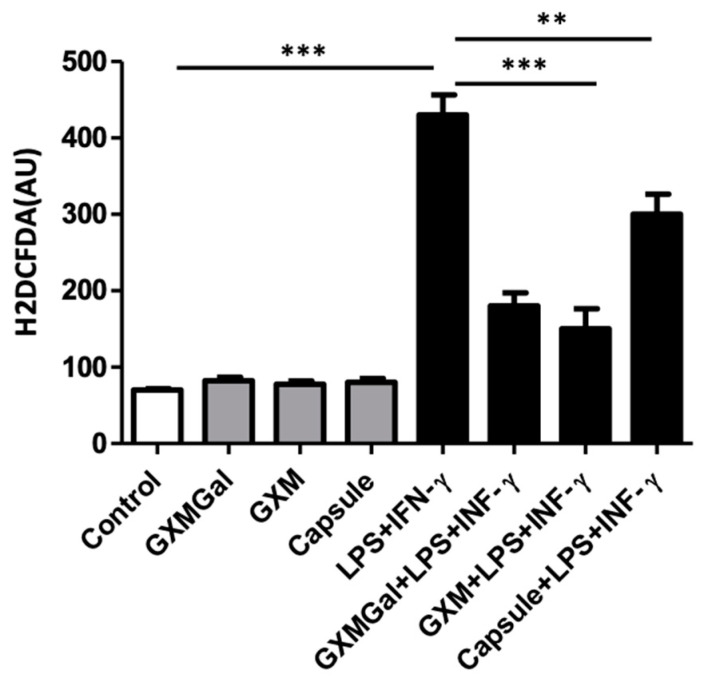
Reactive species of oxygen (ROS) production by DH82 cells treated with purified capsular polysaccharides from *C. neoformans*. DH82 canine macrophages were cultured (5 × 10^4^/mL) and incubated with H2DCFDA, followed by washing. The cells were treated with GXMGal, GXM, or capsule (100 μg/mL), and some cultures were stimulated with LPS (400 ng/mL) and IFN-γ (1.5 ng/mL) for 24 h. Control represents unstimulated samples that were not treated with polysaccharides. The data represent the mean ± SEM of three independent experiments, conducted in triplicates. Statistical analysis was performed by variance (ANOVA), followed by Dunnett’s post-test. We considered significant differences indicated between ** *p* < 0.01 and *** *p* < 0.001.

DH82 macrophages were cultured in the presence of purified polysaccharides or the total capsule of *C. neoformans* and subsequently incubated with a fluorescent probe that measures the intracellular ROS concentration. The results were obtained after 24 h of incubation (Figure 4).

Surprisingly, we observed that GXMGal, GXM, and the total capsule were capable of inducing a significant decrease in ROS production in cells activated with LPS and IFN-γ. This observation suggests that this pathway was strongly modulated in the presence of fungal polysaccharides, potentially compromising the ability of macrophages to kill yeast.

### 3.5. GXMGal Purified from C. neoformans Modulates the Expression of MHC Class II Molecules

An additional method for assessing the impact of polysaccharide stimulation on DH82 cells is through the evaluation of the surface molecule expression, particularly cellular markers associated with antigen presentation, such as MHC Class II (MHC-II) [24]. DH82 macrophages were cultured and incubated with GXMGal, GXM, or the total capsule, with or without additional stimulation with LPS and IFN-γ, to assess the MHC-II molecule expression using flow cytometry.

**Figure 5:**
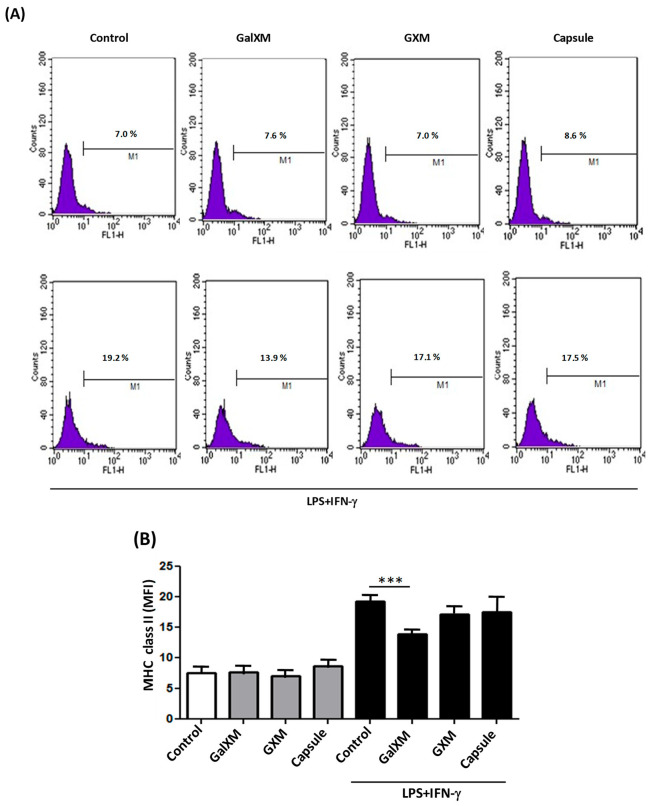
Measurement of MHC class II expression in canine macrophages DH82 treated with polysaccharides from *C. neoformans*. DH82 macrophages (5 × 10^5^/mL) were treated with GXMGal, GXM, or capsule (100 μg/mL). Some cultures were stimulated with LPS (400 ng/mL) and IFN-γ (1.5 ng/mL). Control represents unstimulated samples that were not treated with polysaccharides. After 24 h, cells were prepared for analysis by flow cytometry. M1 marker shows percentage of cells identified by MHC class II (**A**,**B**). Data represent mean ± SEM of three independent experiments, conducted in triplicates. Statistical analysis was performed by variance (ANOVA), followed by Dunnett’s post-test. We considered significant differences indicated by *** *p* < 0.001.

Our findings indicated that polysaccharides did not alter the MHC-II expression in non-activated cells. However, in the presence of additional LPS and IFN-γ stimuli, GXMGal induced a statistically significant reduction in the expression of these molecules (Figure 5A,B). These results suggest that cells that are exposed to the capsular polysaccharides of *C. neoformans* do not exhibit changes in MHC-II expression. However, upon receiving an activating stimulus, they appear to lose the ability to respond at full capacity.

### 3.6. Purified Polysaccharides from C. neoformans Alter DH82 Macrophage Cytokine mRNA Expression

Previous studies have demonstrated that *C. neoformans* polysaccharides can modulate the production of pro- or anti-inflammatory cytokines in murine macrophages and dendritic cells [11,19]. Similarly, we assessed the impact of polysaccharides and the total capsule on the mRNA expression of pro-inflammatory cytokines, such as IL-12 and IL-6, as well as the mRNA expression of the anti-inflammatory cytokine TGF-β.

DH82 macrophages were cultured for 24 h in the presence of GXMGal, GXM, and the total capsule, with or without stimulation with LPS and IFN-γ, and the cytokine’s mRNA expression was quantified using the RT-PCR technique from the lysate of the cultures.

**Figure 6:**
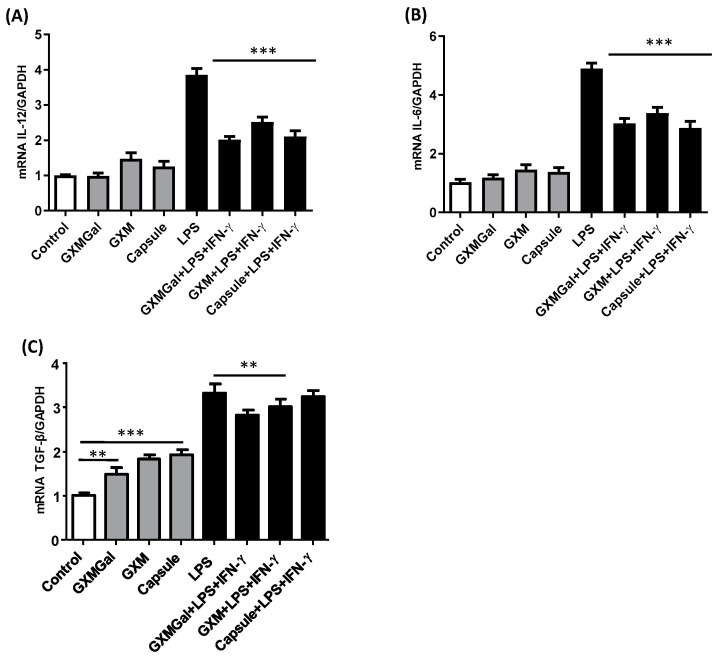
Cytokine mRNA expression of the canine macrophage DH82. Cytokine profile in canine macrophage. DH82 cells (1 × 10^6^/mL) were plated and treated with GXMGal, GXM, and capsule (100 μg/mL). Some cultures were stimulated with LPS (400 ng/mL) and IFN-γ (1.5 ng/mL). Control represents unstimulated samples that were not treated with polysaccharides. After 24 h of incubation, the cells were processed, and mRNA for the cytokines was quantified by qRT-PCR. (A) mRNA expression of IL-12, (**B**) mRNA expression of IL-6, (**C**) mRNA expression of TGF-β. Data from the experiments were normalized using GADPH primers as an endogenous control. The data represent the mean ± SEM of three independent experiments, conducted in triplicates. Statistical analysis was performed by variance (ANOVA), followed by Dunnett’s post-test. We considered significant differences indicated between ** *p*< 0.01 and *** *p* < 0.001.

Our analysis revealed alterations in the mRNA expression of IL-12, IL-6, and TGF-β in DH82 macrophages treated with the polysaccharides (Figure 6A–C). In non-stimulated cells, IL-12 and IL-6 mRNA expressions remained unchanged. However, upon stimulation with LPS and IFN-γ in the presence of polysaccharides, we observed a decrease in the IL-12 mRNA expression by 1.9-fold with GXMGal, 1.5-fold with GXM, and 1.8-fold with the total capsule (Figure 6A). Similarly, a 1.6-fold decrease in IL-6 mRNA expression was observed in stimulated macrophages treated with GXMGal, 1.4-fold with GXM, and 1.7-fold with the total capsule (Figure 6B).

Interestingly, the TGF-β mRNA expression increased by 1.4-fold with GXMGal, 1.8-fold with GXM, and 1.9-fold with the total capsule in unstimulated macrophages. However, a reduction in mRNA expression was observed in stimulated cells, with a decrease of 1.2-fold with GXMGal, 1.1-fold with GXM, and 1.2-fold with the total capsule (Figure 6C).

These findings suggest that contact with the capsular polysaccharides of *C. neoformans* could negatively modulate the production of pro-inflammatory cytokines in DH82 cells, potentially directing them towards an anti-inflammatory profile that could contribute to the persistence of yeasts inside macrophages.

### 3.7. Purified Polysaccharides from C. neoformans Modulate ERK Phosphorylation and PPAR-γ Expression

To assess the impact of polysaccharide or total capsule treatment from *C. neoformans* on intracellular signaling in DH82 cells, we examined the phosphorylation of the ERK protein kinase, a crucial factor in macrophage activation processes. Additionally, we investigated whether the expression of the nuclear factor PPAR-γ could be influenced by capsular polysaccharides, as certain nuclear factors may be associated with suppressive effects on macrophages [25,26].

To investigate whether the modulation of ERK phosphorylation or PPAR-γ expression could account for the observed inhibition of key microbicidal activities in macrophages, such as phagocytosis and the expression of pro-inflammatory cytokines, we conducted assays with GXMGal, GXM, or total capsule in the presence or absence of LPS and IFN-γ for 24 h. Subsequently, the protein extracts were analyzed using the immunoblot technique.

**Figure 7:**
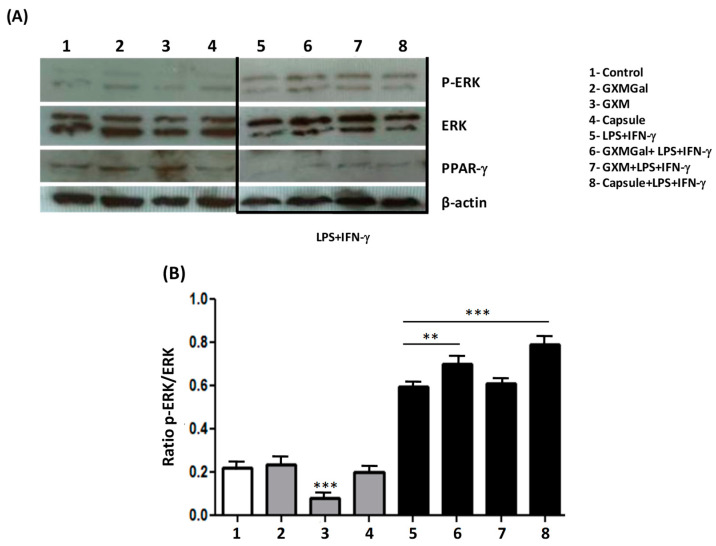

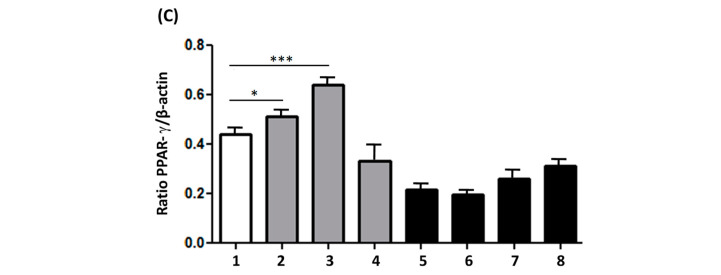
MAPK phosphorylation and PPAR- γ activation. Canine macrophages DH82 (5 × 10^5^) were plated and treated with GXMGal, GXM, and capsule (100 μg/mL). Some cultures were stimulated with LPS and IFN-γ. Control represents unstimulated samples that were not treated with polysaccharides. After 24 h of incubation, whole-cell lysates were loaded onto SDS-PAGE gels. The blot was run and probed with the following antibodies: (**A**) anti-phospho ERK antibody, (**B**) anti-PPAR-γ antibody and (**C**) anti-PPAR-γ. The same blots were then stripped and reprobed with antibodies to non-phosphorylated proteins to determine absolute protein levels. Bar graphics show the ratio of phosphorylated and total proteins. The band densitometry of Western blotting was analyzed using the Scion Image software 4.0.3.2. The data represent the mean ± SEM of three independent experiments, conducted in triplicates. Statistical analysis was performed by variance (ANOVA), followed by Dunnett’s post-test. We considered significant differences indicated between * *p* < 0.05, ** *p* < 0.01 and *** *p* < 0.001.

In terms of ERK phosphorylation, our findings revealed that unstimulated macrophages exhibited a significant decrease in ERK phosphorylation in the presence of GXM. Conversely, when DH82 cells were activated with LPS and IFN-γ, ERK phosphorylation increased in the presence of GXMGal and total capsule (Figure 7A,B). This suggests that non-activated DH82 cells may be negatively modulated by GXM, while activated cells in contact with polysaccharides enhance ERK-dependent signaling.

In an opposing trend, the expression of PPAR-γ showed an increase in DH82 macrophages in the presence of GXM and GXMGal (Figure 7A,C), indicating that these polysaccharides may steer DH82 cells towards an inhibitory profile, potentially facilitating the persistence of the fungus within the organism.

## 4. Discussion

Different models have been extensively studied to reveal the mechanisms of interaction between host cells and parasitic agents [27,28,29]. For many infectious agents, the type of interaction with host cells can determine the resolution or success of the infection. Among the host cells, macrophages stand out for their importance in innate and adaptive immunity [8,29]. *C. neoformans* is an infectious agent that is capable of replicating within macrophages, as reported by several authors [30,31,32]. Furthermore, it has been demonstrated that macrophages could be used by *C. neoformans* as a Trojan horse to transport yeast to strategic points in the body, such as the CNS [7,33].

Although several studies point to the relevance of the interaction of macrophages with *C. neoformans*, no study has been carried out with the aim of understanding the effect of the interaction of the capsular components of *C. neoformans* with canine macrophages. In this work, we used the canine macrophage DH82 cell line, and our group has demonstrated the importance of this cell line in the interaction with parasites as a replacement method [34,35]. Using DH82 canine macrophages treated with purified *C. neoformans* polysaccharides, we demonstrated for the first time their immunomodulatory effects. Previous work has shown that purified polysaccharides from *C. neoformans* can lead to the death by apoptosis of murine macrophages [11]. Thus, we first treated canine macrophages DH82 with different concentrations of GXMGal, GXM, and total capsule, with an incubation of 24 h, to see if the polysaccharides would be able to induce apoptosis in canine macrophages. Surprisingly, it was not possible to observe apoptosis induction at any of the concentrations used. These data suggest that macrophages from different species may show variations in resistance to apoptosis effects resulting from contact with capsular polysaccharides.

The ability to bind and phagocytose infectious agents is a fundamental biological function for macrophages. However, it is known that components of the *C. neoformans* capsule inhibit phagocytic activity and may compromise efficient elimination by this mechanism [20,36]. To assess whether purified polysaccharides would reproduce this effect, we incubated canine macrophages DH82 with GXMGal, GXM, and the whole capsule and used *S. cerevisiae* yeasts. We observed that the polysaccharides were able to inhibit the ability of macrophages to bind to yeasts, as well as phagocytose. The polysaccharides indistinctly led to a decrease in phagocytic activity. We observed a decrease in the number of yeasts attached to macrophages, the number of cells that phagocytosed, and the number of phagocytosed yeasts. These data corroborate findings from other authors, who previously showed that the *C. neoformans* capsule can inhibit phagocytosis by macrophages through various mechanisms [37,38,39].

Based on the observation that GXMGal, GXM, and the total capsule of *C. neoformans* influenced phagocytosis, we investigated whether phagocytosed *S. cerevisiae* yeasts would undergo fungicidal action by DH82 macrophages by counting the CFUs of recovered yeasts after phagocytosis. Experiments performed on murine macrophages [20] suggest that vesicles secreted by *C. neoformans* influence the amount of CFU produced by recovered yeasts after being phagocytosed. In our experiment, the high number of yeast colonies recovered from cells treated (100 µg/mL) with polysaccharides suggests a relationship with a modulating action of the microbicidal activities of the macrophage by the polysaccharides, which is consistent with previous work that showed inhibition of the production of pro-inflammatory cytokines [11] and can be extrapolated to the physiological model, where the impairment of macrophages is related to the inhibition of the immune system leading to a greater fungal burden [4,5,11,17,40]. The presence of numerous CFUs in macrophages with polysaccharides suggests that they decrease the macrophage’s fungicidal activity.

Despite the reduced rate of phagocytosis in the presence of the capsular polysaccharides of *C. neoformans*, it was shown that the yeast that was phagocytosed by these macrophages remained viable and able to grow. For this reason, we analyzed a microbicidal mechanism of oxidant production by phagocytes. ROS play a crucial role in protection against fungal infections, as observed in patients with chronic granulomatous disease (CGD), who are very susceptible to fungal infections [41,42]. Furthermore, the decrease in ROS production may compromise the differentiation of T lymphocytes to the Th1 profile, which is the protective profile [43]. ROS are a factor that participates in the inflammatory process observed in canine cryptococcosis and is associated with excessive granuloma formation [3,44,45]. Our results show that polysaccharides were able to inhibit ROS production in DH82 macrophages, suggesting that the inhibitory effect on ROS production may have favored yeast survival.

After phagocytosis, within the phagosome, yeasts are under the influence of low pH, ROS, reactive nitrogen species, and nutrient deprivation [46]. These challenges are neutralized by yeast through powerful mechanisms. Phagocytosed yeasts can upregulate the gene expression of oxidative stress enzymes, starvation responses, and the autophagic machinery [47,48]. In a model of NADPH oxidase-null mice, cryptococcal infection was contained, and the fungal load in both the brain and lung was decreased [43], suggesting that inflammatory ROS was also prejudicial to the host.

Although ROS act in the death of fungi, this usually happens in a pro-inflammatory environment [49]. In our model, where canine macrophages treated with polysaccharides showed ROS inhibition, this suppressor environment points to the important role of ROS in the microbicidal activity of DH82 canine macrophages.

Macrophages play a crucial role in the processing and presentation of antigens, which is essential for the adaptive immune response. The expression of MHC molecules, particularly MHC-II, is crucial, because it upregulates macrophages, acting as antigen-presenting cells (APCs) and facilitating the activation of lymphocytes, leading to an adaptive immune response [50]. Previous studies have demonstrated that rat macrophages exposed to *C. neoformans* decrease the expression of MHC-II and costimulatory molecules, as well as human dendritic cells in the presence of capsular polysaccharides [51,52]. Impairment in the expression of these molecules can prevent the activation of T lymphocytes and a consequent protective response [19]. DH82 macrophages treated with capsular polysaccharides did not increase the basal expression of MHC-II, contributing to the non-responsiveness of the immune system imposed by *C. neoformans* on the host [53]. The basal expression of MHC-II presented in our results is similar to that found by other groups [54,55,56]. DH82 canine macrophages stimulated with LPS and IFN-γ increase the expression of MHC-II, representing a known ability of these factors to positively regulate the expression of this surface molecule [55,56]. However, a statistically significant decrease in MHC class II expression induced by the presence of GXMGal was observed for the first time, indicating a possible suppression of the canine macrophage DH82’s response, mediated by this polysaccharide that was purified from C. neoformans; however, previous data only characterized the involvement of GXM in this mechanism of action [17]. However, it is known that *C. neoformans* induces the expression of MHC-II in glial cells when in contact with the fungus [57].

Macrophages produce cytokines that mediate and direct the immune response to infection. In cryptococcosis, the crucial role of this function has been demonstrated, with the polarization of macrophages, through the cytokine profile, influencing the course of infection by *C. neoformans* [58]. We evaluated the cytokines IL-12 and IL-6, which help protect the host, and TGF-β, which may contribute to the persistence of the pathogen. While IL-6 contributes to differentiation towards the Th1 profile, which is considered a protective profile, TGF-β inhibits the differentiation of Th1 and Th2 profiles and contributes to the differentiation of Treg cells [59,60].

Our results show that GXMGal, GXM, and the total capsule modulate the mRNA expression of IL-12, IL-6, and TGF-β. Although non-stimulated DH82 macrophages do not alter the mRNA expression of IL-12 and IL-6 cytokines, we clearly saw that when stimulated, these cells exhibited a significant reduction in the mRNA expression of IL-12 and IL-6. On the other hand, TGF-β had its mRNA expression increased in non-stimulated cells and reduced in response to stimuli. This modulation in cytokine mRNA expression suggests that the increase in TGF-β in initial moments of contact with capsular polysaccharides may compromise the production of pro-inflammatory cytokines in canine DH82 macrophages. The inhibition of pro-inflammatory cytokines such as IL-12, which is fundamental in autocrine signaling in the activation of macrophages [61], as well as the inhibition of IL-6, can be determinant not only for macrophage activation but also in the induction of the adaptive response [62]. Thus, the action of polysaccharides may compromise the activation of macrophages. Previous works have demonstrated the anti-inflammatory role that is predominantly exerted by GXM [11,53]. However, our results suggest that, for canine macrophages DH82, GXMGal, GXM, and the total capsule exert the same inhibitory effect on the mRNA expression of the evaluated cytokines.

The results presented so far led to the profile of canine DH82 macrophage suppression. Therefore, we investigated the intracellular signaling in the face of stimulation with the capsular polysaccharides of *C. neoformans*. The results obtained show that GXMGal, GXM, or the total capsule do not induce ERK phosphorylation, which occurs in the presence of LPS and IFN-γ. We also observed a decrease in phosphorylation in the presence of GXM, showing the suppressive characteristic exerted by these polysaccharides, since ERK is part of the large family of MAPK that are involved in several cellular mechanisms, including proliferation and cell activation upon stimulation [63,64]. Another intracellular marker investigated was PPAR-γ, a nuclear factor that is activated in the presence of suppressive stimuli [25,65]. Based on the action that LPS and IFN-γ induce in macrophages, we observed through an increased expression of PPAR-γ that capsular polysaccharides, especially GXM polysaccharide, induce the suppression of DH82 canine macrophages.

**Figure 8:**
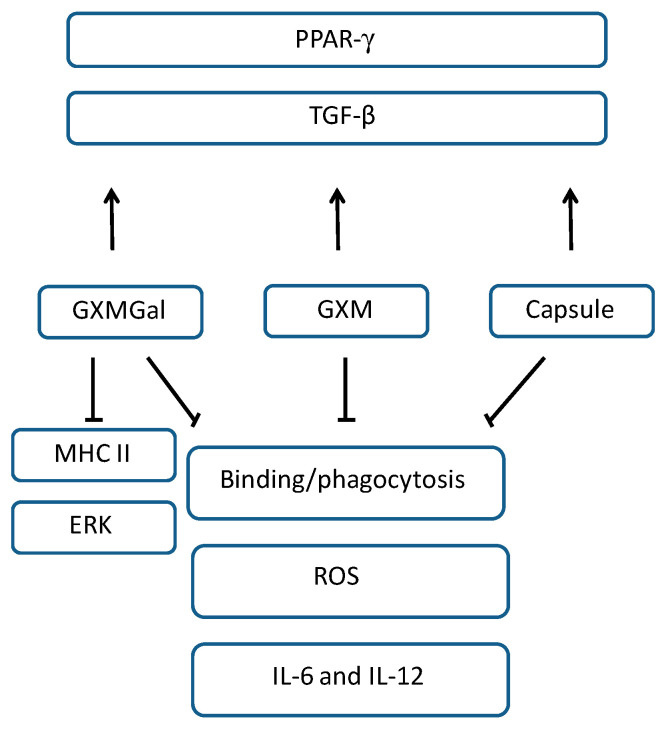
Modulating effects of *C. neoformans* capsular polysaccharides on DH82 canine macrophages. GXMGal, GXM, and capsule inhibit binding and phagocytosis, ROS production, and IL-6 and IL-12 mRNA expression. GXMGal inhibits the expression of MHC II and ERK. TGF-β and PPAR-γ’s mRNA expressions were increased in the presence of GXMGal, GXM, and total capsule.

All aspects evaluated in the present study, such as decreased phagocytosis, inhibition of ROS, IL-12 and IL-6 mRNA expression, increased TGF-β mRNA expression, combined with decreased MHC class II expression, suggest that polysaccharides lead to the impairment of fundamental microbicidal activities of macrophages. In addition, the evaluation of intracellular signaling revealed that in the presence of polysaccharides, there is a reduction in ERK phosphorylation and increase in PPAR-γ expression, reinforcing the suppressor aspect exerted by the polysaccharides on canine macrophages DH82, and these findings are summarized in Figure 8. These data can contribute to the understanding of the impairment of the microbicidal activities of macrophages that could favor the permanence and dissemination of the fungus in the canine host.

However, we understand that all the data presented here deserve to be verified in the future using macrophages differentiated from canine monocytes obtained from blood. Additionally, it is important to assess the dosage of cytokines produced by these cells in response to the stimuli. This further investigation will provide a more comprehensive understanding of the interactions between *C. neoformans* polysaccharides and macrophages in canine hosts, shedding light on potential therapeutic strategies or preventive measures against cryptococcosis.

## 5. Conclusions

The present study suggests a suppressive role of *C. neoformans* capsular polysaccharides in DH82 canine macrophages. Through this suppression profile observed in canine macrophages, it can be deduced that the fungus can persist within the macrophage, potentially leading to systemic infection. Macrophages exhibiting signs of suppression, such as reduced phagocytosis, inhibition of ROS production, decreased mRNA expression of pro-inflammatory cytokines, and increased PPAR-γ expression, meaning that the infection could disseminate to various host sites, including the CNS.

This study represents the first demonstration of the immunomodulatory role of purified capsular polysaccharides from *C. neoformans* in canine macrophages. We recognize the importance of carrying out more detailed future assessments on the interaction of purified capsular polysaccharides and DH82 cells. Furthermore, we understand that the lack of studies with primary cells is a limitation, so additional confirmation of these findings in canine macrophages obtained from peripheral blood is essential.

## Data Availability

The data are contained within this article.
